# Household Hints and Recipes

**Published:** 1876-07

**Authors:** 


### Household Hints & Recipes.

PASTE FOR TIN.—A solution of shellac in naptha or alcohol may be used for fixing labels on tin.

---

DIAMOND CEMENT.—For glass or china, is nothing more or less than isinglass boiled in water to the consistency of cream, with a small portion of rectified spirit added; it must be warmed when used.

---

TO REMOVE WAX SPOTS.—To take wax spots out of cloth or silk, drop spirits of turpentine or alcohol on the spot; then with a sponge rub it gently, repeating the process until the spot disappears.

---

CEMENT.—A cement impermeable to air and steam, and especially well adapted to use for gas or steam pipes, is made of powdered graphite 6 parts, slaked lime 3 parts, sulphate of lime 8 parts, and boiled oil 7 parts, well kneaded.

---

IVORY—TO RENDER DUCTILE.—Ivory may be made ductile so as to be worked into any desired form by soaking it in a solution of pure phosphoric acid; exposure to the air hardens it, but it may be made pliable again by immersion in hot water.

---

JELLY WATER.—This is excellent for fever patients or those suffering from summer complaint:

One large teaspoonful of wild cherry or blackberry jelly;
One goblet of ice-water.
Beat up well.

---

A HINT FOR THE LAUNDRY.—The following is from *Harper's Bazar:* A tablespoonful of black pepper put in the first water in which gray and buff linens are washed will keep them from spotting. It will also keep the colors of black or colored cambrics or muslins from running, and does not harden the water. A little gum arabic imparts a gloss to ordinary starch.

---

LOTIONS FOR FRECKLES.—The following are both good and harmless cosmetics:—

(1.) Borax, 3 grs.
Rose water, 5 dr.
Orange flower-water, 5 dr.

(2.) Orange flower-water, 1 pint.
Glycerine, 1 oz.
Borax, 1 dr.

HOW TO REMOVE STAINS FROM STEEL KNIVES.—The very best way to clean a stained steel knife is to cut a solid potato in two, dip one of the pieces in brick-dust, such as is usually used in knife-cleaning, and rub the blade with it.

---

CONDENSED EGGS.—A factory has lately been established at Passau, in Bavaria, to work a process for supplying the nutrition of eggs in a condensed form. The method adopted seems to be merely that the eggs are dried and then reduced to a fine meal. This is packed into air-tight tins, and thus a supply of the most complete food is provided in the smallest possible compass.

---

FOR FROST-BITE.—Being very appropriate to the winter season, we may note that Dr. Kepes, the surgeon of the Austrian North Pole expedition, states that he found excellent effect in frost-bite from the following mixture:

℞
Iodine, 4 parts.
Ether, 30 parts.
Collodion, 100 parts.
By weight.

---

MUCILAGE FOR MINERALOGICAL SPECIMENS.—Mr. F. C. Hill sends to the *American Journal of Pharmacy*, a recipe for mucilage to mend fossils and minerals. The cement is described as invaluable, being very adhesive, never becoming brittle or scaling off, and answering well for gumming labels, &c. Starch, two parts; white sugar, eight parts; gum arabic, sixteen parts; water, sufficient. Dissolve the gum, add the sugar and starch, and boil until the starch becomes transparent.

---

TO REMOVE GREASE SPOTS.—In the removal of grease from clothing with benzine or turpentine, people generally make the mistake of wetting the cloth with either of these liquids, and then rubbing it with a sponge or piece of cloth. In this way the fat gets dissolved, but spreads over a greater space without being removed; the benzine or turpentine evaporates, leaving the fat behind on a greater surface than before. The only way really to remove a grease-spot, is to place soft blotting-paper beneath and on top of it, the spot to be thoroughly saturated with benzine before covering it with blotting-paper; it is then well pressed, best with a warm iron; the fat gets both dissolved and absorbed by the paper. If necessary, this operation may be once or twice repeated until the spots are entirely removed from the clothing.—*Popular Health Almanac.*

TO POLISH TINS.—First rub your tins with a damp cloth; then take dry flour and rub it on with your hands; afterwards take an old newspaper and rub the flour off, and the tins will shine as well as if half an hour had been spent in rubbing them with brick-dust or powder which spoils the hands.

———

CEMENT FOR MARBLE AND ALABASTER.—Mix 12 parts of Portland cement, 6 parts of slaked lime, 6 parts of fine sand, and 1 part of infusorial earth, and make up into a thick paste with silicate of soda. The object to be cemented does not require to be heated. It sets in twenty-four hours, and the fracture cannot be readily found.

———

GAS FROM PETROLEUM.—The manufacture of a barrel of petroleum into a fixed gas can be accomplished at a much less cost than into refined oil. Numerous works for this purpose are now in operation, and more are being erected, while the cheapness with which they can be built and operated puts gas light within the reach of the smallest communities, instead of confining it to the cities, as is and must be the case with coal gas.—*Anthracite Record.*

———

WATERPROOF PAINT FOR CANVAS.—The following is a cheap and simple process for coating canvas for wagon-tops, tents, awnings, &c. It renders it impermeable to moisture, without making it stiff and liable to break. Soft soap is to be dissolved in hot water, and a solution of sulphate of iron added. The sulphuric acid combines with the potash of the soap, and the oxide of iron is precipitated with the fatty acid as insoluble iron soap. This is washed and dried, and mixed with linseed oil. The addition of dissolved India rubber to the oil improves the paint.

———

WHITEWASH.—The following recipe for whitewash is recommended by the Treasury Department to all lighthouse keepers. It answers for wood, brick, or stone. Slake about half a bushel of unslaked lime with boiling water, keeping it covered during the process. Strain it, and add a peck of salt, dissolved in warm water, three pounds of ground rice put in boiling water and boiled to a thin paste, half a pound powdered Spanish whiting, and one pound clear glue, dissolved in warm water; mix these well together, and let the mixture stand for several days. Keep the wash thus prepared in a kettle or portable furnace, and when used put it on as hot as possible, with either painters' or whitewash brushes.

COATING FOR BLACKBOARDS.—

Take 65 parts 95 per cent. alcohol, or
67 " 90 " "
8 " bleached shellac.
8 " Paris black.
4 " elutriated pumice.
½ " Paris blue.
4 " burnt umber.
8 " dryer.

The pigments and pumice are carefully stirred into the alcohol, and the shellac added last.

———

GOULAND'S LOTION.—This preparation is much used in England as a cosmetic, for sun-burn, freckles, tan, and eczema. It is made as follows:

Bitter almonds, 3 oz.
Distilled water, 16 oz.
Corrosive sublimate, 1½ grs.
Sal ammoniac, 2 dr.
Alcohol 4 dr.
Cherry laurel water, 4 dr.

Blanch the almonds and grind them with the water, and pass through a cloth. Dissolve the salts in the cherry laurel water and alcohol. Mix the two solutions.

———

SILVERING GLASS.—D. C. Chapman in *The Scientific American*, recommends the following modification of a known process:

"Having had occasion to silver some small plates of glass, I tried several formulas, and found that the following works very finely, giving a heavy deposit by a single application:

"No. 1. Reducing solution: In 12 ozs. of water dissolve 12 grains Rochelle salts, and boil. Add, while boiling, 16 grains nitrate of silver dissolved in 1 oz. water, and continue the boiling for 10 minutes more, then add water to make 12 ozs.

"No. 2. Silvering solution: Dissolve 1 oz. nitrate of silver in 10 ozs. water; then add *liquor ammonia* until the crown precipitate is nearly but not quite all dissolved; then add one ounce alcohol, and sufficient water to make 12 ozs.

"To silver: Take equal parts of Nos. 1 and 2, mix thoroughly, and lay the glass, face down on the top of the mixture while wet, after it has been carefully cleaned with soda and well rinsed with clean water.

"Distilled water should be used in making the solutions. About two drachms of each will silver a plate two inches square. The dish in which the silvering is done should be only a little larger than the plate. The solution should stand and settle two or three days before being used, and will keep good a long time."

CURRY POWDER.—The London *Chemist and Druggist* says: "We had the following recipe from a native Indian, and we believe it to yield a fine product. The powders must be of the best quality:—

Coriander, fresh seed, roasted but not burnt, and finely powdered, 2 lbs.
Tumeric, fresh root, ditto, ditto, ¾ lb.
Fenugreek, fresh seed, ditto, ditto, 8 ozs.
Mustard seed, ditto, ditto, 8 ozs.
Cummin seed, ditto, ditto, ¾ lb.

To this add cayenne pepper according to taste."

---

CURE FOR LOVE OF LIQUOR.—At a festival at a reformatory institution, recently, a gentleman said, of the cure of the use of intoxicating drinks: "I overcame the appetite by a recipe given to me by old Dr. Hatfield, one of those good old physicians who do not have a percentage from a neighboring druggist. The prescription is simply an orange every morning half an hour before breakfast. 'Take that,' said the doctor, 'and you will neither want liquor nor medicine.' I have done so regularly, and find that liquor has become repulsive. The taste of the orange is in the saliva of my tongue, and it would be as well to mix water and oil as rum with my taste."

---

PAINTING OLD BUILDINGS.—An inexpensive but durable method of painting old buildings is as follows: First give them a coat of crude petroleum, which is the oil as it comes from the wells, and which can be procured for four or five dollars per barrel. Then mix one pound of "metallic paint," which is brown or red hematite iron and finely ground, to one quart of linseed oil, and apply this over the petroleum coat. The petroleum sinks into the wood, and makes a ground-work for the iron and oil paint. The color of the iron paint is a dark reddish brown, and is not at all disagreeable; it is very durable, and is fire-proof.—*Manufacturer and Builder.*

---

WATER-PROOF GLUE.—One ounce of gum sandarac and one ounce of mastic are to be dissolved together in a pint of alcohol, to which an ounce of white turpentine is to be added. At the same time a very thick glue is to be kept ready, mixed with a little isinglass. The solution of the resins in alcohol is to be heated to boiling in a glue pot, and the glue added gradually with constant stirring, so as to render the whole mass homogeneous. After the mixture is strained through a cloth, it is ready for use, and is to be applied hot. It dries quickly and becomes very hard, and surfaces of wood united by it do not separate when immersed in water.—*Jour. of App. Chem.*

ANOTHER.—A glue which will resist the action of water is made by boiling one pound of glue in two quarts of skimmed milk.

---

FRUIT EATING.—A London journal remarks: "When fruit does harm, it is because it is eaten at improper times, in improper quantities, or before it is ripened and fit for the human stomach. A distinguished physician has said that if his patients would make a practice of eating a couple of good oranges before breakfast, from February till June, his practice would be gone. The principal evil is that we do not eat enough of fruit; that we injure its finer qualities with sugar; that we drown them with cream. We need the medicinal action of the pure fruit in our system, and their cooling, corrective influence."

---

COPYING INK REQUIRING NO PRESS.—Take one ounce of coarsely broken extract of logwood, and one drachm of crystallized carbonate of soda; heat in a porcelain vessel with eight ounces of distilled water, until the solution is of a deep red color. Then remove from the fire and stir in one ounce of glycerine, fifteen grains of neutral chromate of potash dissolved in a little water, and a mucilaginous solution of two drachms of finely pulverized gum arabic. This ink keeps well, and does not attack steel pens. The impression is taken on thin, moistened copying-paper, at the back of which is placed a sheet of writing-paper.

---

GILDING.—Place in a plate, leaf gold, add a little honey, stir the two substances carefully together with a glass stopper, the lower end of which is very flat. Throw the resulting paste into a glass of water mixed with a little alcohol; wash it and leave it to settle. Decant the liquid, and wash the deposit again. Repeat the same operation until the result is a fine, pure, and brilliant powder of gold. This powder mixed with common salt, and powdered cream-of-tartar, and stirred in water, serves for gilding. As another method of gilding, Boulet Mouvel gives the following: Dissolve in *aqua regia* one gram of fine gold, previously rolled out very thin, in a porcelain capsule, heated on the sand bath, and concentrated till it is the color of ox blood. Add a pint of distilled water, hot, in which have been dissolved four grams of white cyanide of potassium. Stir with a glass rod, and filter the liquor through unsized paper.

To gild with this liquid, it is heated a little above lukewarmness, and the articles to be gilt are immersed in it and supported on a piece of very clean zinc.

---

TO FURBISH OIL CLOTHS.—A correspondent in the *Scientific American* gives the following: "Dissolve 2½ pounds paraffin in 1 gallon oil of turpentine, by the aid of a gentle heat, and apply with a sponge or piece of flannel, while warm. Let it remain on the oil-cloth twenty-four hours; then polish with flannel. This solution not only renovates but preserves the cloth. I have used it on oil-cloths which have been down four years, and they look as good as new. The same preparation may also be used on painted floors. When rubbed with flannel, it will have a beautiful gloss, equal to varnish."

W. L. T. says: To cleanse articles from tar, rosin, or any compounds of a resinous character, the use of flax-seed meal, moistened with water is recommended.

---

TO REMOVE GREASE AND OTHER SPOTS.—The following recipes are based on different principles, the one to remove the spots by dissolving the grease, the other by first acting on it chemically:

Take of Benzine, 20 oz.
Alcohol (strong), 5 oz.
Ether, 2 drs.
Ammonia, 1 dr.

JAVELLE WATER.—

Take of bleaching powder, 1 oz.
Carbonate of potassa, 1 oz.
Water, 33 oz.

Triturate the bleaching powder in the cold with 25 oz. of water, then add the carbonate of potassa, previously dissolved in the rest of the water, shake well and let it settle. The supernatant liquor is filtered if necessary, and mixed with one ounce of hydrochloric acid, when it is ready for use.—*Druggist's Circular.*

---

SCOURING LIQUID.—For a considerable time Panama Wood and Panama Extract have been of great use in France. The following is the recipe given by M. Leclerc for what he calls the *Esprit de Panama*, for scouring and removing grease from tissues of all kinds and worn clothes. To take out spots the liquid is used pure, but for general scouring it is mixed with four or five times its own quantity of water:

In 22 gallons of hot water, dissolve white Marseilles soap 15½ lbs., and carbonate of potash 1.3 lbs. or 15 or 18 lbs. of soft soap. To the solution add extract of Panama 1.1 lbs.; then in another vessel mix ox or sheep gall 15 quarts, and ammonia at 22°, 3 pints. Heat this mixture, skim it, let it cool, and then add alcohol at 90°, 3.3 gallons; decant and filter.

Take one third part of the soap mixture and two third parts of the gall mixture, and add some aromatic essence.

---

FEEDING BABIES WITH STARCH.—There are certain very simple principles in dietetics which many people are as "slow to learn" as some of the equally elementary laws of hygiene to which we referred in last No. For instance, they cannot get it through their skull that starch, though a valuable article of food in combination with other things that supplement its deficiencies, cannot of itself furnish all the nutriment needed by a growing infant. This is clearly and pointedly "put" by Dr. Henry McCormac, in the London *Sanitary Record*, as follows:

The nurse or the fond mother boils a quanity of some starchy matter, previously well blended with the spoon, and, pleased with its bland flavor and slab aspect, administers it forthwith to her darling baby. She wonders why the baby does not thrive better, but is unaware that the very commonest and coarsest food would, in all essentials, be preferable. Arrowroot, any kind of starch, corn-flour so-named, but in fact, starch, is inferior in all respects to well-boiled oatmeal porridge or groat gruel. It cannot compare with wheaten meal, or even rice porridge; it is inferior to boiled barley or any of the preparations of Indian corn, and also to the common potato. What beautiful children have I not seen, again and again, in Irish cabins, whose food was the potato, with possibly a little oatmeal porridge and buttermilk. The coarsest, plainest substance, if only coupled with fresh air by night, plenty of out-door exercise by day, cleanliness, and warmth, is preferable to the richest fare without.

Starches of any kind are bereft of the ingredients essential to animal life. In strictness, a portion of those ingredients is present; but where are the phosphates for example, without which the bones cannot be framed? If it were not for the milk, which happily proves the vehicle of the starch or fecula, the infant would inevitably perish.

Once for all, starch food, though it may answer very well as food for adults, as a portion of mixed diet, is quite unfit *per se*, as an exclusive infant diet. It is not worth belly room, and ought invariably to give place to the foods I have named.—*Jour. Chem.*

